# Blunted Reward Responsiveness During Social Feedback: Associations with Peer Victimization and Anhedonia in Socially Anxious Adolescents

**DOI:** 10.1002/brb3.71581

**Published:** 2026-07-28

**Authors:** Corinne N. Carlton, Ligia Antezana, John A. Richey

**Affiliations:** ^1^ Department of Psychology and Human Development Vanderbilt University Nashville Tennessee USA; ^2^ Department of Psychology Virginia Tech Blacksburg Virginia USA; ^3^ Department of Psychiatry University of Pittsburgh School of Medicine Pittsburgh Pennsylvania USA

**Keywords:** adolescence, anhedonia, peer victimization, reward, social anxiety, social stress

## Abstract

**Background:**

The present pilot study aimed to (1) Characterize neural markers of reward sensitivity during periods of social stress; (2) Evaluate clinical relations between neural reward markers and anhedonia; and (3) Investigate if peer victimization was associated with ventral striatum (VS) suppression and anhedonia symptoms during social stress.

**Methods:**

A total of 28 adolescents between the ages of 13 and 17 (*M*_age_ = 15.31; *SD* = 1.51; 55.2% cisgender girls) were included in analyses and completed a semi‐structured interview, self‐report questionnaires regarding social anxiety, stress, depression, and anhedonia, and a magnetic resonance imaging (MRI) scan while engaging in the Island Getaway task. VS BOLD signal activation estimates were then extracted during discrete phases of the task (e.g., anticipation of social feedback and outcome of social feedback) and statistically compared within‐subjects.

**Results:**

Results revealed that when in the presence of social stress (defined as the potential for negative feedback), socially anxious adolescents demonstrated significantly suppressed VS activation relative to feedback anticipation. Additionally, the reduced VS activation during the outcome phase was significantly correlated with anhedonia. Moreover, relational peer victimization was associated with suppressed VS activation.

**Conclusions:**

Findings identify novel mechanisms associated with anhedonia and blunted reward processing in socially anxious youth that could be improved via interventions that target positive‐valence systems.

## Graphical abstract

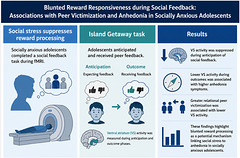

## 1 Introduction

Adolescence coincides with normative increases in fear of negative social evaluation (Westenberg et al. 2007), as well as clinically significant forms of social anxiety, including social anxiety disorder (SAD; American Psychiatric Association 2013; Farrell et al. 2019; Solmi et al. 2021). During the adolescent developmental period, SAD ranks among the most prevalent anxiety disorders (at roughly 10%–15% prevalence rate; Kessler et al. 2005; Merikangas et al. 2010) and is linked to significant functional impairment across educational and occupational domains (e.g., Moitra et al. 2011; Vilaplana‐Pérez et al. 2021). SAD has been further associated with lower self‐reported quality of life in both adolescent and adult samples and follows a chronic course if left untreated (Albano and Hayward 2004; Ollendick et al. 2014; Park et al. 2021). Social anxiety during adolescence also causes significant impairment within interpersonal domains. For example, in a recent systematic review, de Lijster and colleagues (2018) concluded that adolescents with SAD experience more peer victimization, less peer acceptance, and increased levels of loneliness on average as compared to those without SAD. Although prior work has primarily examined neural correlates of SAD maintenance in adults (e.g., Becker et al. 2017; Richey et al. 2017), considerably less is known about the neural vulnerabilities that may potentiate and maintain social anxiety symptomatology during *adolescence*. Accordingly, the purpose of the present study is to evaluate a novel, theory‐driven mechanism of social anxiety maintenance, focusing on social reward processing in socially anxious adolescents.

Broadly, adolescence is characterized by heightened sensitivity to social reward (Somerville 2013). Prior work has also illustrated that acute psychological stress suppresses reward processing during outcome phases (Lincoln et al. 2019). This stress‐induced suppression of reward processing may reduce motivational drive and therefore represent a risk factor for the development of anhedonia (i.e., diminished interest or pleasure in previously rewarding stimuli), which may in turn maintain social anxiety symptoms by diminishing motivation to engage in social behaviors (see Richey et al. 2019 for theoretical review). Reward‐processing deficits associated with anhedonia have been observed across development; however, accumulating evidence suggests that these effects are not uniform across age groups. Meta‐analytic findings indicate that blunted neural responses during reward processing, particularly within striatal regions, are more robust and consistently observed in adolescent samples relative to adults (Keren et al. 2018). This developmental specificity is notable given that adolescence is characterized by heightened sensitivity within reward‐related neural systems (Galván 2010; Somerville 2013), as well as increased salience of social evaluative contexts. Together, these features underscore adolescence as a critical period for examining reward‐related vulnerability processes, particularly in the context of social stress and the emergence of internalizing symptoms such as anhedonia.

### 1.1 Social Anxiety and Reward Processing

Reward neurocircuitry typically refers to the meso‐limbic dopamine (DA) pathway (Haber and Knutson 2010). This pathway consists of several interconnected brain regions, including the ventral tegmental area, ventral striatum (VS), orbitofrontal cortex, ventromedial prefrontal cortex, and the anterior cingulate cortex (Haber and Knutson 2010). Collectively, these areas are sensitive to both the magnitude and probability of non‐social (i.e., monetary) as well as social reward (Rademacher et al. 2010; Saddoris et al. 2015; Schultz 1998, 2000). Moreover, reward network activation is also distinguishable according to the temporal phase of processing, involving both anticipatory (i.e., expecting a reward) and consummatory (i.e., receiving a reward) periods (Rademacher et al. 2010). Prior empirical studies have broadly demonstrated that socially anxious adults show blunted neural activity across multiple reward regions during anticipation of social reward, suggesting that adults with SAD may demonstrate a relative lack of motivational preference for social reward (e.g., Cremers et al. 2015). The VS is of particular interest, as it has been consistently implicated as a marker of blunted reward processing during anticipation of social reward in adults with SAD (Becker et al. 2017; Richey et al. 2017).

Whereas empirical work among adults with SAD has consistently supported the notion of blunted reward processing in key reward regions of the brain, similar work in adolescents has paradoxically revealed heightened sensitivity to reward among adolescents with or at risk for social anxiety symptoms (for review see Richey et al. 2019). Adolescence is characterized by heightened sensitivity to reward in general (see Casey et al. 2015; Schreuders et al. 2018) and social evaluative reward in particular (Somerville 2013). Empirical work has indicated that adolescents at risk for SAD (i.e., exhibited heightened behavioral inhibition during early childhood) demonstrate heightened activation in the VS to monetary reward as compared to controls (Bar‐Haim et al. 2009; Guyer et al. 2012; Guyer et al. 2014). Theoretical advances, particularly the Sensitivity Shift Theory (Richey et al. 2019), emphasize the VS as a central node in a developmental feedback loop that links social stress to altered reward learning and anhedonia. This framework proposes that heightened VS sensitivity in adolescence may initially reflect an adaptive or compensatory response to social evaluative contexts, which over time may contribute to the development of maladaptive avoidance and diminished reward anticipation—hallmarks of SAD. Importantly, this theory highlights the VS as a mechanistic nexus where reward valuation, social stress, and risk for anhedonia converge, making it a critical target for empirical examination in socially anxious youth.

### 1.2 Impact of Social Stress on Reward Function

Theoretical work has posited that adolescence is a critical period for the development of normative patterns of reward reinforcement. In typical development, adolescents have been shown to recruit significantly greater VS activity in response to reward in general as compared to adults and more broadly demonstrate a higher sensitivity to reward than adults (e.g., Ernst et al. 2009; Galvan et al. 2006; see Galván 2010 for review). This sensitivity to reward, and social reward in particular, provides a generally adaptive role in normative development (see Somerville 2013). On the other hand, enhanced neurobiological sensitivity to reward, combined with potentially adverse environmental factors (e.g., social stress and peer victimization), may maladaptively lead to negative future expectations about social reward by potentiating the impact of failed coping outcomes (i.e., suppressing motivated action to resolve future aversive emotions). Given the general increased salience to social reward demonstrated in adolescents (Somerville 2013), the experience of peer victimization may represent a particularly impactful social stress experience. Indeed, peer victimization experiences have been linked to maladaptive emotional functioning and social anxiety in youth (De Los Reyes and Prinstein 2004; La Greca et al. 2005). Further, peer victimization has been shown to be associated with lower VS activation in youth during win phases of reward tasks (see Dobbelaar et al. 2025), suggesting that peer victimization may be an important factor to consider in social stress‐reward paradigms. It has been further theorized that this pattern of frustrative non‐reward may further lead to the development of clinically significant anhedonia, with adolescence serving as a distinct risk period (Carlton et al. 2020; Richey et al. 2019). This stress‐induced suppression of reward processing could serve as a specific and pernicious combination of factors that explain the development of anhedonia among socially anxious adolescents.

In adult samples, prior empirical work has indicated that the presence of stress suppresses reward circuitry function and that repeated exposure to stress is indeed related to the development of anhedonia (e.g., Bogdan and Pizzagalli 2006; Pizzagalli 2014). However, little work has investigated this relation in socially anxious adolescent samples. Prior work has shown that following acute social stress, healthy adolescents demonstrate blunted VS response during the outcome phase of a reward task (Lincoln et al. 2019)—a marked shift from the hypersensitivity generally displayed by adolescents in rewarding contexts (see Somerville 2013). In fact, in at least one study, younger children who are at risk for developing SAD (based on temperamental factors such as elevated behavioral inhibition and heightened wariness) have shown heightened neural processing to threats during anticipation of social stressors (Jarcho et al. 2019). Although this study did not examine regions of reward, this provides initial evidence in youth samples that exposure to a social stressor may potentiate brain‐based sensitivity during anticipation phases of social stress that could be akin to patterns seen in adult SAD samples. Collectively, this work, in combination with the heightened fear of negative evaluation and rejection that is central to SAD, suggests that in the presence of social stress (which tends to significantly increase during adolescence; Eiland and Romeo 2013), exposure to the potential for social rejection, as a form of acute stress, may have a blunting effect on reward processing in socially anxious adolescents. Moreover, this potential blunting effect may be present in both anticipatory and outcome phases of reward processing, with theoretically stronger blunting occurring during anticipation of reward receipt (Richey et al. 2019).

### 1.3 Current Study

The present pilot study aimed to accomplish the following within a sample of socially anxious adolescents (ages 13–17): First, to characterize markers of reward sensitivity during periods of social feedback using a well‐validated social feedback paradigm. Based on prior work showing blunted patterns of reward responsiveness in youth following exposure to an acute social stressor (Lincoln et al. 2019) and work demonstrating that youth at risk for SAD also demonstrated altered neural processing in the context of anticipating peer evaluation (Jarcho et al. 2019), we expected that the VS would demonstrate blunted patterns of activation during the anticipatory phase of social feedback stress (as evidenced by aggregate blood‐oxygen‐level‐dependent (BOLD) levels during the built‐in delay period of the task that occurs immediately prior to social feedback receipt) as compared to outcome phases and that the degree of VS suppression during anticipation of social feedback will correlate with social anxiety symptoms. Second, to evaluate clinical relations between fMRI reward markers and anhedonia. We expected that suppressed anticipatory VS activation would be associated with higher anhedonia symptoms. Third, to investigate if elevated levels of prior exposure to stress (i.e., peer victimization) are associated with the degree of VS suppression and anhedonia symptoms in a social feedback context. We hypothesized that higher levels of peer victimization would predict anticipatory VS suppression and anhedonia presentation.

## 2 Methods

### 2.1 Participants

All participants provided informed parental consent and adolescent assent to participate in the present study. The current study was approved by the IRB (#22‐011). To be eligible to participate in the proposed study, all individuals had to be between the ages of 13 and 17 years old, meet cutoff scores of at least 29.5 on the Liebowitz Social Anxiety Scale for Children and Adolescents (Masia‐Warner et al. 2003), had to indicate the presence of moderate stress by scoring at least a 14 on the Perceived Stress Scale‐10 (Cohen and Williamson 1988), and had to be right‐handed (see Jang et al. 2017). Adolescents must have met a high social anxiety and moderate perceived stress threshold to be included in the present study to represent a sample of adolescents who might be more “at‐risk” for the development of anhedonia based on sensitivity shift theory (Richey et al. 2019). Adolescents who were actively suicidal and/or who experienced restrictions barring fMRI (e.g., metal in the body, history of seizures, and claustrophobia) were excluded from the study. A total of 30 adolescents between the ages of 13 and 17 years old (*M*_age_ = 15.31; *SD* = 1.51) participated in the present study (see Table 1 for demographics); however, a total of 28 adolescents had usable data for analyses.

**TABLE 1:** Demographic information.

Age (mean [SD])	15.31 (1.51)
**Family income (mean range)**	$65,000–$100,000
**Family employment**	
Full‐time	75.9%
Part‐time	13.8%
Unemployed	6.9%
Did not answer	3.4%
**Gender**	
Cisgender girl	55.2%
Cisgender boy	24.1%
Non‐binary	13.8%
Transgender boy	6.9%
**Race**	
White	86.2%
Black	6.9%
Asian/Pacific Islander	6.9%
**Sexual orientation**	
Heterosexual	41.4%
Homosexual	10.3%
Bisexual	20.7%
Pansexual	10.3%
Queer	13.8%
Other	3.5%

### 2.2 Procedures

Adolescents and their parents completed an online screener and remote eligibility session to confirm inclusion and exclusion criteria prior to an in‐person session. Next, participants underwent the semi‐structured interview and were scheduled for their in‐person session. During the in‐person session, participants were provided an overview of the in‐person session (e.g., that they would be answering some questions and then playing a game with other peers while in the MRI), and then consent/assent was re‐established. Then, participants filled out self‐report measures related to anhedonia, social anxiety, perceived stress, and depression to get baseline levels of these constructs. Participants also filled out an additional MRI safety screening form to assess for any changes in eligibility status prior to entering the MRI scanner and were asked to have a photo taken of themselves by the experimenter to set up their online profile for the game. Participants were told that their peers would be viewing their profiles. Following completion of all self‐report measures, participants received instructions on how to respond in the Island Getaway task (Kujawa et al. 2014; see below and in Supporting Information for more information) and completed a practice trial of the task. Of note, participants practiced the task one additional time prior to playing while they were in the scanner to ensure comfort and comprehension. Following the completion of the scan, participants filled out a questionnaire regarding their impression of the game. Lastly, participants were fully debriefed about the artificial nature of the social rejection they may have experienced to alleviate any potential increased stress responses.

### 2.3 Measures

#### 2.3.1 Semi‐Structured Interview

##### 2.3.1.1 Anxiety Disorders Interview Schedule for DSM‐5 Child Version (ADIS‐C)

The ADIS‐C is a semi‐structured interview that assesses for internalizing (e.g., major depressive disorder [MDD], SAD, and generalized anxiety disorder [GAD]) and externalizing disorders (e.g., attention‐deficit/hyperactivity disorder, oppositional defiant disorder, and conduct disorder) (Silverman and Albano 1996, 2016). For the purposes of the present study, only select modules relevant to internalizing psychopathology of the ADIS‐C were administered to the adolescent and parent conjointly based on recommendations from prior work (Carlton et al. 2024; Radtke et al. 2023). Specifically, the SAD, GAD, and MDD modules of the ADIS‐C were administered to gain a diagnostic picture of functioning with regard to key internalizing disorders. No externalizing modules were administered in the present study. Presence or absence of a diagnosis did not impact eligibility. However, active suicidality was an exclusionary criterion for the present study.

#### 2.3.2 Self‐Report Questionnaires

##### 2.3.2.1 Liebowitz Social Anxiety Scale for Children and Adolescents (LSAS‐CA Masia‐Warner et al. 2003)

The LSAS‐CA is a 24‐item measure that assesses social anxiety severity in youth. Participants were asked to indicate first how anxious or fearful they would be in certain situations (e.g., “Giving a verbal report or presentation in class”; “Telling others that you disagree or that you are angry with them”) on a 4‐point scale ranging from 0 = *I do not fear it at all* to 3 = *Severely fear it*. Next, they were asked to rate how often they avoid those situations on a 4‐point scale ranging from 0 = *Never avoid it* to 3 = *Usually* (*like 67%–100% of the time*). Participants were considered eligible if they met a cutoff score of 29.5 on the LSAS‐CA, indicating that they are experiencing significant current social anxiety. Previous work has demonstrated that the LSAS‐CA has shown excellent psychometric properties (*α* = 0.83–0.95 across subscales) and that a score of 29.5 is optimal for distinguishing SAD from other anxiety disorders in adolescent samples (Masia‐Warner et al. 2003). The LSAS showed excellent internal consistency in the current study (*α* = 0.95).

##### 2.3.2.2 Perceived Stress Scale‐10 (PSS‐10; Cohen and Williamson 1988)

The PSS‐10 is a 10‐item self‐report measure that determines how much participants perceive situations in their lives to be stressful. Participants were asked to indicate how often they have felt a certain way (e.g., “How often have you felt nervous and stressed?”; “How often have you felt that things were going your way?”) on a 5‐point scale ranging from 0 = “*Never*” to 4 = “*Very Often*.” Participants were considered eligible if they met a cutoff score of at least 14, indicating that they had been experiencing at least moderate stress over the last month. The PSS‐10 has been administered among diverse populations of adolescents (e.g., Carlozzi et al. 2010) and has demonstrated good internal consistency (*α* = 0.82). The PSS demonstrated good internal consistency in the present study (*α* = .078).

##### 2.3.2.3 Patient Health Questionnaire for Adolescents (PHQ‐9A; Johnson et al. 2002)

The PHQ‐9A is a 9‐item questionnaire that measures depression symptom presentation in adolescents. Participants were asked to indicate how often they have been bothered by each symptom over the past 2 weeks on a scale ranging from 0 = “*Not at all”* to 3 = “*Nearly every day*.” The PHQ‐9A has consistently demonstrated good psychometric properties in adolescent samples (Johnson et al. 2002; Richardson et al. 2010). The PHQ‐9A showed good internal consistency (*α* = 0.83). It should also be noted that item nine regarding suicidality was dropped for administrations of the PHQ‐9A such that suicidality was not assessed.

##### 2.3.2.4 Snaith‐Hamilton Pleasure Scale (SHAPS; Snaith et al. 1995)

The SHAPS is a 14‐item measure that assesses anhedonic experiences. Participants were asked to rate how strongly they agree with each item (e.g., “I would enjoy being with my family or close friends”; “I would find pleasure in the scent of flowers or the smell of a fresh breeze or freshly baked bread”) on a 4‐point Likert scale from 0 = “*Strongly disagree”* to 3 = “*Strongly agree*.*”* The SHAPS has consistently demonstrated good psychometric properties in both adult and adolescent samples (Leventhal et al. 2006; Leventhal et al. 2015; Snaith et al. 1995). The SHAPS demonstrated adequate internal consistency (*α* = 0.76).

##### 2.3.2.5 Peer Experiences Scale Revised (RPEQ; De Los Reyes and Prinstein 2004)

The RPEQ is a 20‐item questionnaire that measures experiences with peer victimization in the domains of overt, relational, reputational, and prosocial contexts. Participants were asked to report how frequently they have experienced certain situations (e.g., “A teen made other people not talk to me” and “A teen damaged something of mine on purpose”) on a 5‐point scale ranging from 1 = “*Not at all*” to 5 = “*A whole lot*.” Higher scores on the RPEQ indicate more frequent experiences of peer victimization. The RPEQ has shown excellent psychometric properties (De Los Reyes and Prinstein 2004). The RPEQ was administered at screening in the present study and demonstrated good internal consistency (Overt *α* = 0.91, Relational *α* = 0.82, Reputational *α* = 0.89, Prosocial *α* = 0.84).

#### 2.3.3 fMRI Task

##### 2.3.3.1 Island Getaway Task (Kujawa et al. 2014)

The Island Getaway Task is a social feedback task where participants must vote on which people should stay or get kicked off of a virtual island. Participants were told that they would be playing a game with 13 age‐matched peers that are participating at the same time in other labs and were first asked to enter information about themselves (e.g., name, age, and interests) to compile a profile for themselves, including a photo of them taken by the experimenter. Across all rounds, participants were asked to vote on whether each co‐player should “stay” or be “voted off” the island. There is a built‐in delay prior to receiving feedback to increase believability. Following this delay, participants were shown the co‐player's votes on them as to whether the co‐players thought that the participant should stay in the game (i.e., the outcome phase of the task). They received either positive feedback (i.e., co‐player voted for them to stay), negative feedback (i.e., co‐player voted for them to be kicked off), or neutral feedback (i.e., no feedback). During the practice trials, participants are told that when a yellow square appears on the screen “no feedback was received due to a computer network error” or “due to a co‐player not voting in time” (Kujawa et al. 2014). All participants “survived” the game by making it to the “Big Island” with a group of peers, but acceptance, rejection, and neutral feedback were presented in equal proportions across the task (21 trials per condition). Participants completed six rounds of the task.

Most prior work using the Island Getaway task has evaluated reward responsiveness via electroencephalogram (EEG). Through this work, the Island Getaway task has consistently and reliably elicited event‐related potentials (ERPs) modulated by social reward in large community samples of children (Kujawa et al. 2014; Kujawa et al. 2017). Additionally, the Island Getaway task has shown moderate stability over the course of 3 years with the reward‐related positivity—an ERP that indexes reward activation—being reliably enhanced for social feedback (Kujawa et al. 2018). Moreover, in addition to the Island Getaway task eliciting a reward response across adolescent samples, confederate peer “co‐players” are generally perceived as realistic (Kujawa et al. 2017, 2018), thus adding an additional layer of ecologically valid social context to the present study. See Supporting Information for additional information about the MRI version of this task piloted in the present study.

### 2.4 Data Analytic Plan

#### 2.4.1 Neuroimaging Data Acquisition

Neuroimaging data were collected using a 3.0T Siemens Magentom Trio scanner. To reduce head movement in the scanner, foam cushions were placed around the participant's head. Structural scans were obtained using a rapid gradient echo sequence (repetition time [TR]/echo time [TE]: 2000 ms/3.02 ms; FOV: 22 cm; image matrix: 184 × 256 × 192; voxel size = 0.82 mm × 1.00 mm × 1.00 mm). Functional runs utilized a hyperscan EPI sequence (TR = 2000 ms, TE = 25 ms, flip angle = 90°, 220 mm field of view [FOV], 64 × 64 matrix). Standard orientation of functional slices occurred (i.e., 30° superior‐caudal to the anterior and posterior [AC/PC] commissures). Functional images were obtained via an interleaved approach. Axial slices for the functional BOLD signal were 37.4 mm and yielded 3.4 mm × 3.4 mm × 4.0 mm voxels.

#### 2.4.2 fMRI Preprocessing

Skull‐stripping was run via the brain extraction tool for structural images in FSL (Oxford Centre for Functional Magnetic Resonance Imaging of the Brain [FMRIB], Oxford University, UK). All functional data were preprocessed via Statistical Parametric Mapping software (SPM12) leveraged by Nipype, a Python‐based framework for handling neuroimaging data (Gorgolewski et al. 2011; http://nipy.org/nipype). Preprocessing of functional data included slice timing correction. The middle functional image was then corrected using a six‐parameter rigid‐body transformation. Next, functional images were co‐registered to structural images in native space. Structural images were normalized into a standard space (i.e., MNI space; Montreal Neurological Institute) via the use of Advanced Normalization Tools (ANTS; Avants et al. 2011). Via statistical analyses drawn upon in Nipype, we estimated the general linear model (GLM) for BOLD responses in SPM12. All code involved in the fMRI preprocessing and analysis of the present study is included on GitHub in addition to original task code and at https://github.com/daisylabhub/ARSA.

#### 2.4.3 Data Analytic Plan

First, beta weights for bilateral VS were extracted via the use of an anatomical region of interest (ROI)‐driven approach based on the Harvard‐Oxford subcortical atlas.^1^ Bilateral VS regions were extracted and analyzed separately. This approach was selected to preserve sensitivity to potential hemispheric differences in reward‐related activation, as averaging across hemispheres may obscure lateralized effects. Prior neuroimaging research has demonstrated that reward‐related processes may vary across processing stages and task demands and are not necessarily uniformly expressed across hemispheres (X. Liu et al. 2011; Arsalidou et al. 2020). Consistent with prior work examining ventral striatal activation bilaterally (e.g., Knutson et al. 2001; Rademacher et al. 2010), we retained separate estimates for left and right VS in all analyses. The anticipation phase (i.e., all phases of anticipation) versus outcome phase (i.e., all phases of outcome) beta weights in the VS were compared statistically via paired‐samples *t*‐tests. Then, through the use of Nipype as described above, average functional BOLD contrasts were statistically compared between anticipatory (i.e., all times when the participant was anticipating feedback) and outcome (i.e., all times when the participant received feedback) phases via a one‐sample *t*‐test to determine whether the activation patterns of the VS were diminished during social feedback anticipation as compared to social feedback outcomes. Correlations were also assessed between VS and social anxiety severity as well as the VS and anhedonia.

Next, we performed a regression with anticipatory VS BOLD activation as the independent variable and anhedonia as the dependent variable while controlling for age and gender. Moreover, we also ran the same analyses for VS activation during outcome to examine differences between anticipatory activation as it relates to anhedonia as compared to outcome VS activation as it relates to anhedonia.

Additional regression analyses were performed such that levels of peer victimization across domains were inputted as independent variables and VS activation across phases (i.e., anticipatory and outcome) were the dependent variables while controlling for age and gender.

## 3 Results

### 3.1 Descriptive Statistics

Descriptive statistics for all variables of interest are included in Table 2. Of note, one participant was excluded from subsequent analyses due to being an outlier (> 3 SD) with respect to observed parameter estimates in the VS, and one participant was excluded due to excessive motion, resulting in a total sample of 28. Overall, the sample presented as significantly socially anxious, with a mean total score on the LSAS of 69.93, well above the cutoff of the LSAS‐CA of 29.5 as suggested by Masia‐Warner et al. (2003). Additionally, the sample was also in the high stress range per cutoffs on the PSS‐10 (Cohen and Williamson 1988) and demonstrated less anhedonia as compared to depressed adult samples (Rizvi et al. 2015).

**TABLE 2:** Descriptive statistics and correlations for all study measures of interest.

	M (SD)	1	2	3	4	5	6	7	8
1. LSAS	68.59 (26.3)	—							
2. PSS‐10	23.17 (5.65)	0.46*	—						
3. PHQ‐9A	11.34 (4.99)	0.16	0.51**	—					
4. SHAPS	1.83 (2.21)	0.17	0.37*	0.32	—				
5. RPEQ overt	1.33 (0.45)	−0.13	−0.14	0.02	−0.09	—			
6. RPEQ relational	2.17 (0.91)	0.22	−0.03	0.04	0.01	0.38*	—		
7. RPEQ reputational	1.94 (1.20)	0.08	0.16	0.26	0.05	0.46*	0.46*	—	
8. RPEQ prosocial	2.71 (0.96)	−0.06	0.37*	0.21	0.19	0.51	0.09	0.15	—

Abbreviations: LSAS‐CA = Liebowitz Social Anxiety Scale for Children and Adolescents; PSS‐10 = Perceived Stress Scale‐10; PHQ‐9A = Patient Health Questionnaire for Adolescents; SHAPS = Snaith–Hamilton Pleasure Scale; RPEQ = Peer Experiences Scale Revised.

*p < 0.05; **p < 0.01.

### 3.2 Reward Sensitivity during Periods of Social Feedback

**FIGURE 1:**
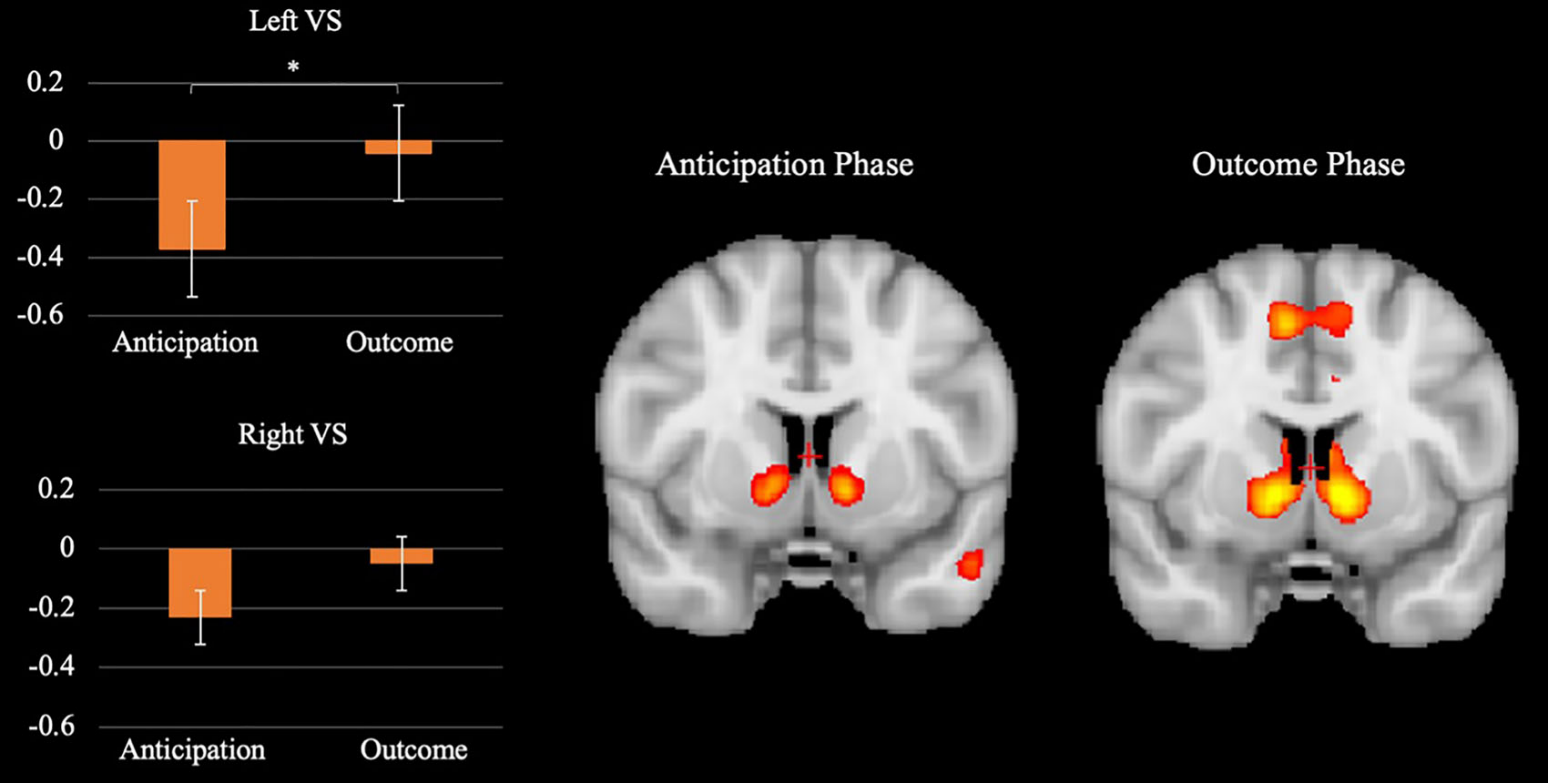
Average VS BOLD activation during general anticipatory and outcome phases.

A paired samples *t‐*test was performed to determine whether the VS BOLD signal during the anticipatory phase of the task was significantly blunted as compared to the outcome phase. Results demonstrated that there were significant differences in the left VS during anticipation versus outcome phases of the task (*t*(27) = −1.84, *p* = 0.039) such that the VS was significantly less active during anticipation as compared to outcome phases of the task. However, when considering right VS, there was no significant difference between anticipation and outcome activation (*p* = 0.190). Results from second‐level functional analyses conducted in Nipype are shown in Figure 1 indicating significant activation patterns in the VS during both phases (FDR corrected at *p* < 0.05; anticipatory phase: left *k* = 457, peak voxel [*x*, *y*, *z*]: 101, 134, 68, *t* = 4.58, right *k* = 480, peak voxel [*x*, *y*, *z*]: 77, 132, 67, *t* = 24.15; outcome phase left *k* = 1858, peak voxel [*x*, *y*, *z*]: 100, 135, 70, *t* = 4.55, right *k* =  *k* = 1315, peak voxel [*x*, *y*, *z*]: 79, 135, 71, *t* = 4.40).^2^ The full cluster tables are provided and listed in Tables S1 and S2.

Correlations were also carried out to assess the association between social anxiety severity and average beta weight VS activation during anticipation as well as outcome phases. Results indicated that left VS activation during the anticipatory phase was not significantly correlated with social anxiety severity (*r =* −0.27, *p* = 0.167), and left VS activation during the outcome phase was also not significantly associated with social anxiety severity (*r* = −0.36, *p* = 0.058). These patterns are consistent when evaluating the right VS, such that activation during the anticipatory phase was not significantly correlated with social anxiety severity (*r* = −0.28*, p* = 0.147), and right VS activation during the outcome phase was also not significantly associated with social anxiety severity (*r* = −0.34, *p* = 0.078).

### 3.3 Clinical Relations between VS Activation and Anhedonia

Regression analyses were run to assess the VS as a predictor of anhedonia while controlling for age and gender. Results indicated that neither the right nor left VS at anticipation or outcome significantly predicted anhedonia (*p*s > 0.05) when controlling for age and gender. However, correlational analyses (not controlling for covariates) indicated that there were no significant associations between anhedonia for both the left and right VS during the anticipatory phase (all *p*s > 0.05). For the outcome phase, both left and right VS demonstrate significant relations with anhedonia (left VS: *r* = −0.39, *p* = 0.040; right VS: *r* = −0.39, *p* = 0.040) via the SHAPS, suggesting that anhedonia may correlate with the strength of reward signal during socially valenced outcomes, though this may be obscured by influence of adolescent gender and age on these constructs.

### 3.4 Association of Peer Victimization With VS Activation and Anhedonia

#### 3.4.1 VS Activation

Additional regression analyses were performed to assess for the association of peer victimization and VS responses. Step 1 variables included age and gender, and step 2 variables included all domains of peer victimization; VS activation was added as the dependent. Results indicated that higher relational peer victimization showed a non‐significant trend‐level association with blunted left VS activation during the anticipatory phase (step 1 *R* ^2^ = 0.040; step 2 *R* ^2^ = 0.239, *t*(27) = 1.86, *p* = 0.077) and in the right VS activation during the anticipatory phase (step 1 *R* ^2^ = 0.133; step 2 *R* ^2^ = 0.315, *t*(27) = 2.06, *p* = 0.052) above and beyond age or gender. This pattern appears to be specific to the anticipatory phases, as no domain of peer victimization was significant or trending significance as a predictor of VS activation during outcome (all *ps* > 0.05).

## 4 Discussion

The overall purpose of this pilot study was to characterize reward processing during anticipatory and outcome phases of social feedback and assess associations between these processes and theoretically relevant constructs (i.e., anhedonia and peer victimization) in socially anxious adolescents when under the condition of acute social stress. The work presented here was collectively motivated by gaps in the literature regarding reward processing in the context of peer social feedback and how stress‐induced suppression of neural responses in the VS may be related to the development of anhedonia within socially anxious adolescents.

Regarding characterizing reward activation during social feedback contexts, it was hypothesized that the VS would demonstrate blunted patterns of activation during the anticipatory phase of social feedback stress as compared to outcome phases based on prior work suggesting that exposure to acute stress blunts anticipatory VS response in adolescents (Lincoln et al. 2019) as well as the idea that socially anxious adolescents view social rejection as a form of stress. Results from the present study indicated that this hypothesis was partially supported, such that left VS activation during anticipation of social feedback was significantly lower as compared to left VS activation during the outcome phase. Moreover, functional analyses also support these findings with an identical pattern of blunted left VS activation during the anticipatory phase. Thus, these results are the first to demonstrate diminished expectancy of reward in social stress contexts for socially anxious youth, supporting prior theoretical work positing this pattern (Richey et al. 2019). It should be noted that this pattern was not observed in the right VS, indicating potential lateralization in anticipatory reward processing. Although the present study did not include a priori hypotheses regarding hemispheric differences, examining left and right VS separately allowed for the detection of potential lateralized effects that may have been obscured by averaging across hemispheres. Prior work suggests that reward‐related processes may exhibit hemispheric specificity depending on task demands and reward phase (Arsalidou et al. 2020). Additionally, meta‐analytic evidence indicates that reward processing involves partially dissociable neural systems across anticipatory and outcome phases (X. Liu et al. 2011), which may differentially engage left and right striatal regions. However, given the exploratory nature of this analysis and the modest sample size, these findings should be interpreted cautiously. It remains unclear whether the observed lateralization reflects meaningful neurobiological differentiation or sample‐specific variability. Future work should directly compare bilateral versus averaged VS signals in larger samples to clarify the role of hemispheric specificity in social reward processing. Results from the current study add to this line of inquiry by suggesting potential left‐lateralized reward‐related striatal responses in socially anxious adolescents.

Based on prior work indicating that adolescents with high social anxiety, or those at risk for developing social anxiety via high behavioral inhibition, tend to demonstrate heightened striatal activation (e.g., Bar‐Haim et al. 2009; Guyer et al. 2012; Guyer et al. 2014) as compared to typically developing peers, it was hypothesized that the degree VS activation during anticipation of social feedback would be significantly related to social anxiety severity. Results demonstrated that VS activation during the anticipatory phase was not significantly correlated with social anxiety severity. This pattern of results was somewhat surprising given prior work; however, this may be due to the sample consisting solely of highly socially anxious participants, thus limiting potential variability of social anxiety severity. Further, this pattern of findings could be due to differences in samples recruited in prior work; for example, Bar‐Haim et al. (2009) recruited youth with high behavioral inhibition but not social anxiety specifically, while the present study assessed socially anxious adolescents with co‐occurring moderate stress. Future work should assess striatal activation during social anticipatory contexts within adolescent samples with variable social anxiety levels and confirm findings for socially anxious youth samples specifically.

Recent work has theorized that socially anxious adolescents may be particularly vulnerable to the development of anhedonia due to a pernicious combination of blunted reward processing in social contexts leading to diminished approach‐related behaviors and exposure to significant social adversity (e.g., peer victimization) during development (Carlton et al. 2020; Richey et al. 2019). However, limited empirical work has examined reward processing, social adversity, and anhedonia development in socially anxious adolescents. We expected that suppressed anticipatory VS activation would predict increased anhedonia given that prior work has linked blunted VS responding to anhedonia across development, although these effects appear to be more robust and consistently observed in adolescent samples than in adults (Borsini et al. 2020; Keren et al. 2018; Stringaris et al. 2015; Wang et al. 2021). The present findings should therefore be interpreted within a developmental framework. Adolescence represents a period of heightened sensitivity to both reward and social evaluative cues (Galván 2010; Somerville 2013). These factors may amplify the impact of social stress on reward‐related neural systems. Within this context, the current findings may reflect early‐stage or context‐dependent alterations in reward processing among socially anxious adolescents, rather than fully consolidated patterns of reward dysfunction. That is, disruptions in ventral striatal signaling may not yet manifest as stable, trait‐like deficits, but instead may emerge selectively under conditions of social stress or during specific phases of feedback processing. When evaluating associations of anhedonia and VS activation, anhedonia was not significantly associated with anticipatory VS activation but was significantly associated with outcome VS activation. These results are consistent with the possibility that reward‐processing alterations in socially anxious adolescents may be especially sensitive to contextual factors, rather than uniformly expressed across reward phases. Future studies should assess this pattern in a longitudinal framework so that anhedonia can be assessed over time instead of in a state‐like manner, as was done in the present study.

Lastly, we hypothesized that higher levels of peer victimization would predict anticipatory VS suppression and anhedonia severity. These hypotheses were grounded in prior empirical work linking the exposure to early adverse experiences (including peer victimization) to altered reward processing (e.g., Rappaport et al. 2019) as well as prior theoretical work proposing the stress‐to‐anhedonia pipeline (Richey et al. 2019) in socially anxious youth. The hypothesis positing that peer victimization would predict anhedonia was largely unsupported. However, results provided some evidence for the hypothesis that peer victimization was associated with VS activation during anticipation. Specifically, findings revealed trend‐level higher endorsement of experiencing relational peer victimization was significantly associated with blunted VS activation during anticipation above and beyond age or gender. This pattern of results did not hold for VS activation during the outcome phases, suggesting a potential link between overt peer victimization and anticipatory reward processing. Future work should expand on this through the investigation of this relation in larger samples. Results here suggest that peer victimization may specifically act on anticipatory systems of reward processing by suppressing anticipated reward value, which may in turn reduce future approach‐related behaviors.

Taken together, findings from the present study identify potential targets for intervention and prevention in socially anxious adolescents that could be modifiable, although that issue awaits future investigation. Specifically, it may be possible that preventing or buffering against the effects of peer victimization through extant interventions may have the indirect effect of modifying reward processing. The findings reported here are particularly important in that context, given that current “gold‐standard” interventions that focus primarily on negative‐valence systems demonstrate low rates of positive treatment outcomes in socially anxious youth (e.g., Ginsburg et al. 2011). Recently, positive‐valence systems (PVS) function in anxious youth have gained some traction, suggesting that dysfunction in PVS (including reward responsiveness) is an important component of youth anxiety (e.g., Sequeira et al. 2022). Additionally, there has been an emphasis on the development of treatments that target positive valence systems in therapeutic treatments, such as the Positive Affect Treatment (PAT; Craske et al. 2016), the Amplification of Positivity Treatment (AMP; Taylor et al. 2020), and Family Promoting Positive Emotions (FPPE; Burkhouse et al. 2023). Individuals who have participated in these trials have demonstrated significant enhancements in positive affect (Burkhouse et al. 2023) and in reward processing following administration of the intervention (Craske et al. 2023; Kryza‐Lacombe et al. 2021). Thus, these treatment approaches may also represent productive avenues for intervention among socially anxious youth. Future work should assess the effectiveness of these interventions in socially anxious adolescent samples, perhaps also in combination with peer victimization prevention‐based programs, such as UTalk (La Greca et al. 2016).

Overall, the present pilot study answers key preliminary questions about reward processing in socially anxious adolescents when under the condition of perceived social peer evaluation stress. Prior foundational work has consistently noted significantly heightened anticipatory VS signal in the presence of reward in adolescent samples in general as compared to adults (see Galván 2010; Somerville 2013 for reviews), in youth at risk for developing SAD as compared to youth not at risk (Guyer et al. 2014), and in socially anxious youth samples as compared to other psychiatric groups (e.g., Guyer et al. 2012). However, the present study demonstrated a different pattern of reward processing within socially anxious adolescents, such that VS signal was significantly blunted during anticipation of social reward as compared to outcome. While those prior studies set the stage for the current study, these studies did not assess reward processing in socially anxious adolescents within the context of socially valenced peer stress. Thus, the present study extends the literature in this area by adding additional study design elements to the underlying framework of prior work. First, the present work added a social valence to the study design to assess patterns of reward processing in a social context, such as prior adult work utilizing the social incentive delay task (e.g., A. Richey et al. 2017). Second, the present study added an additional layer onto this broader social valence context—a peer salience context—and evaluated whether the presence of social stress that tapped into peer salience (vis‐à‐vis the potential for *peer* rejection) demonstrated consistent patterns with prior work in adults. These distinctions in study design may be critical to understanding the opposite pattern of reward responsiveness demonstrated in the present study because during the adolescent developmental period, peer relationships and peer acceptance are particularly salient (e.g., Somerville 2013) and of central concern for socially anxious adolescents due to the core fear of negative evaluation. Consistent with prior models of acute stress‐induced blunting of VS signals (e.g., Jarcho et al. 2019; Lincoln et al. 2019), the present study represents a proof of concept of this stress‐reward blunting model within socially valenced peer contexts within socially anxious youth. Additionally, it should be highlighted that the findings from the present study fit squarely within a high‐risk profile for anhedonia development based on theoretical work positing this pattern of reward dysfunction in the context of early adversity (Richey et al. 2019) and thus may represent the “pre‐anhedonic” phase of development for this population whereby this vulnerability to social stress is exhibited. Taken together, the present study leads to additional lines of inquiry that should be evaluated. Specifically, future research in this area should consider assessing stress‐reward processing pipelines longitudinally within socially valenced tasks that tap into core contributors of SAD and anhedonia to further characterize reward processing patterns in this population under ecologically valid and ecologically relevant conditions.

### 4.1 Limitations and Future Directions

As with all studies, the results presented here should be taken in light of study limitations. First, while recruitment of a relatively robust sexuality‐ and gender‐diverse sample from a rural community was possible, this sample largely consisted of white individuals who also fell within a relatively high family income range. As such, the present study may not generalize to individuals of different racial backgrounds or lower socioeconomic status. This is not trivial, as children who live in under‐resourced areas tend to experience higher rates of adversity during development (Brendgen et al. 2021; Cicchetti and Lynch 1993; Sedlak et al. 2010). Thus, it is critical that future work intentionally recruit these samples to increase generalizability of the present study to samples whom these variables may impact most. Importantly, the findings presented here should be considered within the context of a small sample size. The present study represents a pilot proof of concept study and collected a modest sample of adolescents between the ages of 13 and 17 years old. While we did control for age in the analyses presented here, we were limited given the small sample, and we could not assess age‐related developmental trajectories, which will be important for future work. As such, future studies should collect robust sample sizes to test that the associations found here generalize to larger samples across the adolescent developmental period. Next, while the present study was able to modify the EEG version of the Island Getaway task to an MRI format, not all portions of the task could be carried over without compromising data collection. First, free response items in the task were limited solely to the profile creation phase due to difficulty starting and stopping functional runs in between each round. Next, likability ratings were also limited to the profile creation phase, such that participants only reported on how much they liked a co‐player prior to beginning the game. This limitation is due to concerns of movement, as well as available button options programmed to the scanner via the button box. Thus, continuous ratings of likability of co‐players were not able to be collected. Additionally, this is the first time this task has been employed via fMRI. As such, at this time we lack formal reliability metrics for this task through this method. Future work should assess the psychometric parameters. Lastly, with regard to the task, although standardized pictures of adolescents (via the NIMH‐ChEFS picture set; Egger et al. 2011) were included, these pictures only had a single positive valence (i.e., smiling faces) to remain consistent. Future work should consider switching out mixed valence images to assess voting preferences and reward signal in the presence of negative or neutral peer facial expressions. Additionally, future work should consider the possibility of adding in photos of peers that the participant may recognize (e.g., friends and/or peers at school). While the present study aimed to assess within‐group associations, future work should consider assessing these findings in socially anxious youth as compared to a control group. Finally, the findings should be interpreted in light of the modest analytic sample size for this pilot fMRI study. Although hypothesis‐driven, within‐subject analyses of task‐evoked activation in a priori regions of interest can be adequately powered to detect moderate‐to‐large effects in smaller samples (Desmond and Glover 2002), smaller samples provide limited precision for estimating individual‐differences associations and may yield unstable effect size estimates (Button et al. 2013; Schönbrodt and Perugini 2013). Converging evidence from large‐scale neuroimaging studies further suggests that reproducible brain–behavior associations often require substantially larger samples (Marek et al. 2022; S. Liu et al. 2023). Accordingly, the present findings linking ventral striatal activation with anhedonia and peer victimization should be considered preliminary and hypothesis‐generating. Future research using larger, well‐powered samples will be necessary to establish the reliability and generalizability of these associations.

## 5 Conclusion

In sum, findings from the present study suggest that under social stress (i.e., the potential for negative feedback), socially anxious adolescents demonstrate significantly blunted VS activation when anticipating feedback. Additionally, results indicate that there may be some associations among VS activation during outcomes and anhedonia severity. Lastly, relational peer victimization may be an important factor to consider in the context of blunted striatal activation during anticipation of social feedback. The results identify potentially modifiable mechanisms associated with anhedonia severity and blunted reward processing in socially anxious youth; however, future work should assess these findings in larger samples of socially anxious youth.

## Author Contributions

**Corinne N. Carlton**: conceptualization, writing – original draft, methodology, formal analysis, investigation, writing – review and editing, project administration. **John A. Richey**: conceptualization, formal analysis, methodology, supervision, writing – review and editing. **Ligia Antezana**: conceptualization, writing – review and editing.

## Ethics Statement

This study was approved by the Virginia Tech IRB (IRB; #22‐011).

## Consent

All participants provided informed parental consent and adolescent assent to participate in the present study.

## Conflicts of Interest

The authors declare no conflicts of interest.

## Supporting information




**Supplementary Material**: brb371581‐sup‐0001‐SuppMat.docx

## Data Availability

Data are available upon request.

## Endnotes

1 Bilateral VS masks were extracted via the use of an anatomical region of interest (ROI)‐driven approach based on the Harvard‐Oxford subcortical atlas. Specifically, the ROI included the nucleus accumbens bilaterally, which corresponds to the core component of the VS implicated in reward processing. The mask was thresholded at 50% probability to ensure anatomical specificity and included a total of approximately 276 mm^3^ voxels (left nucleus accumbens: ∼134 mm^3^; right nucleus accumbens: ∼142 mm^3^).

2 FSL cluster coordinates are displayed in voxel space rather the MNI space.
