# Supplementary material for: Blunted Reward Responsiveness During Social Feedback: Associations with Peer Victimization and Anhedonia in Socially Anxious Adolescents
Source: Brain Behav. 2026 Jul 28;16(8):e71581. doi: 10.1002/brb3.71581 (PMC13411298; doi:10.1002/brb3.71581)
Supplement: Supplementary file 1 — Supplementary Material: brb371581‐sup‐0001‐SuppMat.docx [file BRB3-16-e71581-s001.docx]

**Supplemental Material**

***MRI Version of the Island Getaway Task***

The Island Getaway task used in the present study was modified from the original EEG version to be compatible with MRI systems. Notable changes from the original task include the removal of continuous liking ratings in between rounds based on free responses, the lengthening of anticipation and outcome phase blocks to 7 seconds to allow enough time to capture the hemodynamic response, and the adjustment of adding standardized co-player adolescent images from the NIMH-ChEFS Picture Set (Egger et al., 2011). All adjustments were made in collaboration with the author of the original task. Task code and data cleaning scripts for the MRI version of the Island Getaway task can be found at https://github.com/daisylabhub/ARSA.

**Table S1**

*Main Effect of Anticipation Clusters*

| Size (mm^3^) | *t* max | Voxel Space Coordinates | | |
| --- | --- | --- | --- | --- |
|  |  | X | Y | Z |
| 11710 | 5.42 | 24 | 80 | 94 |
| 6092 | 5.59 | 153 | 88 | 115 |
| 789 | 4.16 | 122 | 145 | 67 |
| 480 | 4.15 | 77 | 132 | 67 |
| 457 | 4.58 | 101 | 134 | 68 |
| 337 | 4.03 | 140 | 131 | 45 |
| 178 | 4.00 | 159 | 91 | 58 |
| 143 | 4.21 | 104 | 50 | 19 |
| 135 | 3.86 | 64 | 49 | 16 |
| 112 | 3.45 | 96 | 157 | 73 |
| 63 | 3.39 | 106 | 179 | 98 |
| 44 | 3.34 | 57 | 42 | 34 |
| 44 | 3.34 | 38 | 98 | 64 |
| 36 | 3.27 | 92 | 118 | 68 |
| 29 | 3.69 | 38 | 47 | 79 |
| 20 | 3.37 | 150 | 59 | 69 |
| 18 | 3.44 | 95 | 43 | 28 |
| 10 | 3.17 | 89 | 174 | 101 |
| 1 | 3.11 | 58 | 38 | 39 |
| 1 | 3.16 | 47 | 50 | 116 |

*Note: Clusters significant at p < 0.05.*

**Table S2**

*Main Effect of Outcome Clusters*

| Size (mm^3^) | *t* max | Voxel Space Coordinates | | |
| --- | --- | --- | --- | --- |
|  |  | X | Y | Z |
| 1858 | 4.55 | 100 | 135 | 70 |
| 1354 | 4.65 | 89 | 117 | 70 |
| 1315 | 4.40 | 79 | 135 | 71 |
| 1024 | 4.36 | 37 | 50 | 85 |
| 531 | 4.17 | 82 | 136 | 122 |
| 335 | 3.72 | 118 | 152 | 75 |
| 285 | 4.77 | 34 | 111 | 127 |
| 240 | 4.15 | 99 | 145 | 114 |
| 230 | 3.63 | 114 | 121 | 121 |
| 67 | 3.55 | 64 | 49 | 16 |
| 55 | 3.20 | 98 | 128 | 125 |
| 39 | 3.33 | 116 | 169 | 67 |
| 19 | 3.28 | 150 | 85 | 122 |
| 18 | 3.38 | 105 | 51 | 19 |
| 18 | 3.49 | 140 | 44 | 87 |
| 11 | 3.36 | 20 | 97 | 96 |
| 9 | 3.30 | 60 | 138 | 41 |
| 9 | 3.22 | 96 | 152 | 67 |
| 7 | 3.26 | 96 | 42 | 27 |
| 5 | 3.14 | 59 | 152 | 80 |
| 3 | 3.19 | 41 | 102 | 136 |

*Note: Clusters significant at p < 0.05.*

**Table S3.**

*Clinical Interview Metrics.*

|  | Mean (SD) | Met Clinical CSR Cutoff |
| --- | --- | --- |
| ADIS CSRs |  | |
| *SAD* | 4.28 (1.99) | *N* = 26 |
| *GAD* | 3.13 (1.96) | *N* = 14 |
| *MDD* | 2.17 (2.33) | *N* = 9 |

**_Note:_** _RPEQ = Peer Experiences Scale Revised; LSAS-CA = Liebowitz Social Anxiety Scale for Children and Adolescents; PSS-10 = Perceived Stress Scale-10; PHQ-9A = Patient Health Questionnaire for Adolescents; ADIS C = Anxiety Disorders Interview Schedule for DSM-5 Child Version; CSR = Clinician Severity Rating; SHAPS = Snaith–Hamilton Pleasure Scale._

**Table S4.**

*Hierarchical Regression Predicting Left VS during Anticipation.*

|  | B | SE B | β | *t* | *p* |
| --- | --- | --- | --- | --- | --- |
| Model 1 |  |  |  |  |  |
| (Constant) | 1.511 | 1.925 | — | 0.785 | .440 |
| Gender | -0.050 | 0.114 | -0.085 | -0.436 | .667 |
| Age | -0.114 | 0.123 | -0.181 | -0.926 | .364 |
| Model 2 |  |  |  |  |  |
| (Constant) | 1.875 | 2.011 | — | 0.932 | .362 |
| Gender | -0.040 | 0.120 | -0.070 | -0.336 | .740 |
| Age | -0.209 | 0.130 | -0.332 | -1.614 | .122 |
| RPEQ Overt | 0.915 | 0.668 | 0.297 | 1.370 | .185 |
| RPEQ Relational | 0.481 | 0.259 | 0.425 | 1.856 | .077 |
| RPEQ Reputational | -0.163 | 0.177 | -0.199 | -0.917 | .369 |
| RPEQ Prosocial | -0.254 | 0.209 | -0.267 | -1.216 | .238 |

**Table S5.**

*Hierarchical Regression Predicting Right VS during Anticipation.*

|  | B | SE B | β | *t* | *p* |
| --- | --- | --- | --- | --- | --- |
| Model 1 |  |  |  |  |  |
| (Constant) | 3.827 | 2.138 | — | 1.790 | .086 |
| Gender | -0.088 | 0.127 | -0.129 | -0.692 | .495 |
| Age | -0.250 | 0.137 | -0.339 | -1.822 | .080 |
| Model 2 |  |  |  |  |  |
| (Constant) | 4.093 | 2.216 | — | 1.847 | .079 |
| Gender | -0.078 | 0.133 | -0.115 | -0.588 | .563 |
| Age | -0.373 | 0.143 | -0.506 | -2.609 | .016 |
| RPEQ Overt | 0.908 | 0.736 | 0.252 | 1.234 | .231 |
| RPEQ Relational | 0.588 | 0.285 | 0.444 | 2.059 | .052 |
| RPEQ Reputational | -0.045 | 0.195 | -0.048 | -0.232 | .819 |
| RPEQ Prosocial | -0.220 | 0.230 | -0.198 | -0.956 | .350 |
